# Trajectories of Purpose in Life Following a Dementia Diagnostic Appointment

**DOI:** 10.1093/geroni/igaa057.917

**Published:** 2020-12-16

**Authors:** Matthew Wynn, Catherine Ju, Patrick Hill

**Affiliations:** Washington University in St. Louis, St. Louis, Missouri, United States

## Abstract

Purpose in life has been linked with better well-being and reduced risk for major illness. As such, work has focused on understanding what leads to changes in sense of purpose during adulthood, with a focus on major life events. Receiving a dementia diagnosis is a major life event that could affect purpose in life both for older adults with dementia and their potential caregivers. To examine this issue participants answered questions at two timepoints, before their diagnostic appointment at a specialized memory clinic, and between two days and two weeks after the appointment. Participants provided self-report ratings of sense of purpose and as well as open-ended answers regarding their purpose in life. Data is available from both caregivers and patients and qualitative coding was performed on participants’ open-ended responses. While there was no significant, mean-level change in purpose in life for patients (t = -.14, p = .88) or caregivers (t = .73, p = .46), some participants exhibited reliable change in sense of purpose. Factors underlying individual increases and decreases in sense of purpose following a dementia diagnostic appointment are explored and discussed.

